# Irradiation‐Induced CD62L Shedding in Human Plasmacytoid Dendritic Cells Depends on ADAM17 and p38 MAPK

**DOI:** 10.1111/imm.70194

**Published:** 2026-09-02

**Authors:** Estelle Leplus, Jamila Kacher, Dalil Hannani, Guillaume Vayssiere, Jean‐Paul Molens, Karine Laulanier, Dan Georgess, Véronique Josserand, Philippe Saas, Séverine Planel, Laurence Chaperot, Joel Plumas

**Affiliations:** ^1^ PDC*Line Pharma SAS Grenoble France; ^2^ Université Grenoble Alpes, CNRS, UMR 5525, VetAgro Sup, Grenoble INP, TIMC Grenoble France; ^3^ Université Grenoble Alpes, Inserm U 1209, CNRS UMR 5309, Institute for Advanced Biosciences (IAB), Team Translational Immunology and Immunotherapy in Chronic Disease Grenoble France; ^4^ Recherche et Développement, Etablissement Français du Sang Auvergne‐Rhône‐Alpes Grenoble France; ^5^ TransCure bioServices Archamps France; ^6^ Université Grenoble Alpes, Inserm U 1209, CNRS UMR 5309, Institute for Advanced Biosciences (IAB), Optimal Grenoble France

**Keywords:** active immunotherapy, ADAM17, cell migration, cell‐based vaccine, immunology, L‐selectin, plasmacytoid dendritic cells

## Abstract

Plasmacytoid dendritic cells (PDCs) represent a crucial bridge between innate and adaptive immunity as they can recognise viruses and viral‐infected cells, secrete type I interferon and prime naive T‐cell responses. Like naive T cells, PDCs express CD62L, an essential molecule involved in the trans‐endothelial migration process, and are supposed to travel directly from blood to lymph nodes to prime naive T cells. We developed a therapeutic PDC line model (PDC*line) derived from a patient with blastic PDC neoplasm, and showing high antigen‐presentation capacities. PDC*line‐based vaccine was tested in clinical trials in melanoma and lung cancer. Efficient specific T lymphocyte priming was observed suggesting the efficient migration of injected PDC*line‐based vaccine to lymph nodes. Here, we investigated the mechanisms involved in this migration and its regulation. In humanised mice, our results demonstrated the efficient migration of the PDC‐based vaccine from blood to lymph nodes and its capacity to prime naive T cells. The expression of CD62L was evaluated following irradiation, which was mandatory in the vaccine manufacturing step and possibly important for its efficacy. CD62L was rapidly shed after irradiation of PDC*line cells and primary PDCs but not of NK‐T cells, demonstrating the cell specificity of the mechanism. Our results show that p38 MAP kinase, known to be activated in response to irradiation and ADAM17 metalloprotease are involved in the rapid CD62L shedding. This new pathway could participate to the regulation PDC transmigration through High Endothelial Venules and homing to secondary lymphoid organs in cellular stress conditions.

## Graphical abstract

Human plasmacytoid dendritic cell‐based vaccines efficiently migrate from blood to lymph nodes and prime naive T cells in humanised mice. Irradiation‐induced CD62L shedding, mediated by p38 MAP kinase and ADAM17, is involved in the regulation of PDC migration to secondary lymphoid organs in this model.

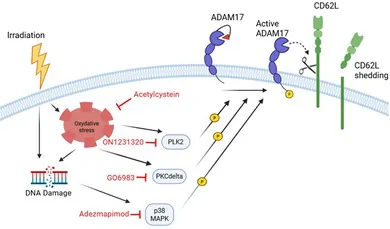

## 1 Introduction

Among dendritic cells, plasmacytoid dendritic cells (PDCs) uniquely bridge innate and adaptive immunity [1, 2]. They play a major role in antiviral immunity by their secretion of type I interferon (IFN‐I) [3]. They are able to recognise and to engulf material from infected cells [4], and once activated, perform efficient antigen cross‐presentation [5].

Like naive T lymphocytes, circulating PDCs express CD62L (L‐Selectin), a crucial molecule involved in the trans‐endothelial migration (TEM) process, suggesting that they could travel directly from blood to lymph nodes (LNs) [6, 7, 8]. During infections by HIV (human), SIV (monkey) or influenza virus (mouse), PDCs leave the blood and accumulate in secondary lymphoid organs/LNs where they produce high levels of IFN‐I, likely entering via high endothelial venules (HEVs) [9, 10, 11, 12, 13]. The direct recruitment of PDCs from blood to LNs across HEVs has been shown in mice, with the demonstration that CD62L was required for PDC attachment and rolling on HEVs [14].

The trafficking of leukocytes requires the coordinated action of adhesion molecules, chemokines and their receptors, among which CD62L whose expression is tightly regulated. Indeed, CD62L contains a membrane‐proximal cleavage site positioned nine amino acids up from the plasma membrane. The ectodomain of CD62L is shed from the cell surface upon neutrophil or T lymphocyte activation leading to its loss after transmigration [15]. This shedding has also been observed during the migration of monocytes through HEVs, demonstrating its role in regulating the polarity in transmigrated monocytes [16]. A disintegrin and metalloproteinase 17 (ADAM17) has often been described as responsible for CD62L cleavage in leukocytes. ADAM17 function is regulated by phosphorylation by intracellular kinases such as protein kinase C (PKC), Polo kinase 2 (PLK2) or p38 mitogen‐activated protein kinases (MAPK) [17, 18]. Besides ADAM17, ADAM8 and ADAM10 are also potential CD62L sheddases [19].

Surprisingly, despite the crucial role supposed for CD62L in the migration of PDCs from peripheral blood to LNs where they can encounter and prime naive T cells, no study has specifically investigated the cleavage of CD62L on the surface of PDCs and the mechanisms involved in this shedding.

CD62L is expressed at the surface of leukaemic PDCs [20, 21], and the GEN2.2 PDC cell line developed from a patient having a blastic PDC neoplasm (BPDCN) displays the same features and represents a valuable tool for the study of normal PDCs function [22, 23]. A good manufacturing practices‐compliant PDC*line cells derived from the same patient was used as a cancer vaccine in phase I/II clinical trials to treat melanoma [24] and lung cancer [25]. In both clinical trials, tumour‐specific T lymphocytes were primed by the tumour‐associated antigens loaded onto the irradiated PDC*line cells, suggesting the efficient migration of injected PDCs to LNs.

The manufacturing process of the PDC*line‐based vaccines comprised an irradiation step that was mandatory during the manufacturing process due to the neoplastic origin of the cells. Ionising radiations are known to induce severe DNA damage due to double‐strand breaks (DSBs). PDCs are able to detect these damage‐associated molecular patterns that can activate Toll‐like receptor 9 or cytosolic DNA sensors such as cGAS–STING, thereby triggering dendritic cell maturation [26, 27], which could participate to the PDC*line vaccine efficacy.

In order to analyse CD62L regulation in PDCs, we studied the priming of naive antigen‐specific CD8+ T cells by PDC*line cells and PDC*line cell migration to LN in vivo in humanised mice. We then examined the expression of CD62L on PDC*line cells and primary PDCs and its modulation by ionising radiation. We finally explored the molecular pathways involved in the regulation of CD62L expression after irradiation, focusing on the role of ADAM sheddase and kinases controlling ADAM activity.

## 2 Materials and Methods

### 2.1 Cell Material

The PDC*line cells used in this study have been extensively described [24, 25]. The cells were cultured in X‐VIVO 15 medium (Lonza, Walkersville, Maryland, USA) in the presence of 10 μg/mL gentamycin (Invitrogen, Thermo Fisher Scientific, Waltham, Massachusetts, USA). When indicated, cells were irradiated using an Actemium/Cegelec BloodXrad 13‐69 X‐ray irradiator.

For biodistribution studies, PDC*line cells were modified using a lentivirus encoding the Luciferase gene using a plasmid (pWPIR‐SFFV‐Luc2‐IRES‐Neo) kindly provided by V. Josserand (Optimal platform, IAB, La Tronche, France).

Peripheral blood mononuclear cells (PBMCs) were isolated from whole blood samples using a centrifugation density gradient (Eurobio Scientific, Les Ulis France). Adult blood was obtained from healthy blood donor volunteers who gave written informed consent in accordance with the Declaration of Helsinki (declared biological collection DC‐2019‐3803, EFS‐AURA). Umbilical Cord Blood units were obtained from the Cell Therapy Unit (EFS‐BFC, Besançon, France) and used for CD34+ stem cell purification according to Transcure bioServices SAS (Archamps, France) standard operating procedures.

### 2.2 In Vivo Priming of Antigen‐Specific CD8+ T Cells

The assessment of CD8+ T cells priming in vivo was performed using NOG (NOD.Cg‐Prkdcscid Il2rgtm1Sug/JicTac) mice humanised with CD34+ human stem cells (huNOG) and vaccinated with peptide‐loaded PDC*line cells. Seven 4‐week‐old female immunodeficient NOG mice (Taconic Biosciences, Ejby, Denmark) were engrafted with HLA‐A*02:01+ CD34+ haematopoietic stem cells derived from umbilical cord blood 2 days after chemo‐myeloablative treatment, in TransCure bioServices facilities. At Week 14, the humanisation rate was 52% on average and greater than 20% (Figure S1). At Week 25, the mice were randomised into two groups and received either Melan‐A‐loaded [28] (10 μM) irradiated PDC*line cells (group 1: 20 million, *n* = 5) or the vehicle (group 2: PBS, *n* = 2) once a week for 4 weeks. Blood samples were collected just before each vaccination and 1 week after the last vaccination (D0, D7, D14, D21, D28). The spleen was removed on the day of sacrifice (D28).

All animal studies described in this study were approved by the local ethics committee (01_TransCurebioServices‐AB‐01) and performed under the authorisation of the French Ministry of Higher Education, Research and Innovation and the Departmental Directorate for the Protection of Populations in Haute‐Savoie (France).

Flow cytometry analyses were performed on an Attune NxT flow cytometer (Life Technologies) after labelling the cells with APC‐anti‐hCD45 and BV421‐anti‐hCD3 antibodies (AB_11153499 and AB_11152082, Beckton Dickinson), PerCP‐Cy5.5‐anti‐hCD8 (AB_2811219, Beckman Coulter), FITC antigen‐specific multimers (Melan‐A/HLA‐A*02:01) from Immudex (Copenhagen, Denmark) and Live/Dead Yellow (L34968, Thermo Fisher Scientific).

### 2.3 Biodistribution Study

The migration of PDC*line cells to LNs was assessed using BALB/c nude mice humanised with human PBMCs and luciferase‐modified PDC*line cells. Seven 7‐week‐old female BALB/c nude mice (CAnN.Cg‐Foxn1nu/Crl from Charles River) received an intravenous injection of 50.10^6^ PBMCs 24 h before being randomly divided into two groups. Mice in groups 1 and 2 received intravenous injections (caudal vein) of irradiated and luciferase‐modified PDC*line cells (*n* = 6) and PBS (*n* = 3), respectively. Four hours later, the mice were sacrificed and the LNs were removed and incubated in a solution containing 300 μg/mL of luciferin reagent before undergoing bioluminescence imaging using a Perkin Elmer IVIS Spectrum device. Results were represented in photons/s/square cm/sr (p/s/cm^2^/sr) and photon/s (p/s).

These experiments were carried out under animal experimentation authorisation obtained from the Ministry of Research and the Ethics Committee (MENSR project authorisation: APAFIS#1870‐2015092413243589 v5).

### 2.4 CD62L Measurement by Flow Cytometry

PDC*line cells were labelled with anti‐CD62L conjugated to FITC (Beckman Coulter, IM1231U) or to APC (Beckman Coulter, B30639) and Live/Dead dye was mixed with antibodies (L34958, Thermo Fisher Scientific).

For experiments on primary PDC, diluted blood samples were treated or not with phorbol‐myristate‐acetate (PMA 5 ng/mL, P‐8139, Sigma Aldrich, positive control) or irradiated at 30 Gy and incubated for 4 or 18 h at 37°C in the presence of IL‐3 (Peprotech, AF‐200‐03) at 50 ng/mL. The cells were then labelled with FITC‐antilineage (Lin) cocktail (BD Biosciences, RRID: AB_400053), BV421‐anti‐HLA‐DR (BD Biosciences, RRID: AB_268742), RB705‐CD11c (BD Biosciences, RRID: AB_3685649), PECy7‐CD123 (BD Biosciences, RRID: AB_400053), Live/Dead Violet (Thermo Fischer Scientific, ref. L34958) and CD62L. CD62L expression was assessed on gated PDCs (Lin^neg^HLA‐DR^pos^CD123^pos^) or T lymphocytes + NK cells population (Lin^pos^HLA‐DR^neg^) by flow cytometry.

For inhibition of ADAMs activity, three chemicals were used alone and in combination: TIMP‐3 (250 nM, R&D systems, 973‐TM‐010), batimastat (1 μM, TOCRIS, 2961) and CGS 27023A (2.5 μM, TargetMol, T3472). Cells were incubated for 1 h at 37°C with the inhibitors, then irradiated or not at 60 Gy and cultured for 2 additional hours at 37°C. For inhibition of oxidative stress, p38 MAPK, PKCdelta and PLK2, PDC*line cells were first incubated for 1 h at 37°C with the following inhibitors from MedChemExpress: acetylcysteine (HY‐B0215), adezmapimod hydrochloride (HY‐10256A), GO 6983 (HY‐13689) and ON1231320 (HY‐100789) and then irradiated or not at 60 Gy and incubated for 1 additional hour at 37°C. Cells were then labelled as previously described.

Cells were acquired using a FACS Canto II flow cytometer Diva (v9.0.1) and analysed using FlowJo (v10.10).

### 2.5 CD62L Measurement in Cell Lysates and Supernatants

PDC*line cells were irradiated and cultured in X‐VIVO 15 medium (1 × 10^6^ cells/mL) for 4 h. The cells were then centrifuged, washed and the resulting pellets were treated for 30 min at 4°C with lysis buffer 17 (R&D Systems, 895943) supplemented with protease inhibitors: aprotinin, leupeptin and pepstatin (TOCRIS, references 4139, 1167 and 1190). Cell lysates were analysed using Proteome Profiler Human sReceptor Array (R&D systems, ARY011) and the proteins were quantified using Pierce BCA Protein Assay Kit (Thermo Scientific, 23 225) according to manufacturer recommendations.

To measure soluble CD62L in supernatants, cells were irradiated and cultured for 4 h in RPMI 1640 with 10% foetal bovine serum (FBS, Life Technologies). Then, cells were centrifuged and the supernatants were collected for measurement of soluble CD62L using the Human L‐Selectin/CD62L DuoSet ELISA kit (R&D Systems, DY728).

### 2.6 Assessment of ADAM8 and ADAM17 Expression

For flow cytometry analysis, irradiated PDC*line cells were labelled with AF488‐anti‐ADAM8 antibodies (R&D Systems, RRID: AB_3654439), AF488‐anti‐ADAM17 antibodies (R&D Systems, RRID: AB_3653806) or the corresponding isotype, IgG1 (R&D Systems, ABVI0419062). Cells were acquired using a FACS Canto II flow cytometer with Diva (v9.0.1) software and analysed using FlowJo (v10.10) software.

To determine mRNA expression levels of ADAM8 and ADAM17, PDC*line cells were irradiated or not and cultured for 2 or 4 h or treated immediately after irradiation. Dry pellets were prepared for RNA extraction using the RNeasy Plus Mini Kit (Qiagen, catalogue no. 74134), and quantification and quality controls were performed using the RNA Nano kit (Agilent, catalogue no. 5067‐1511), Nanodrop and Agilent 2100 bioanalyser. Total RNA was then reverse transcribed using the Superscript VILO IV cDNA Synthesis Kit (Thermo Fisher Scientific, reference 18091050), and quantitative PCR was performed using a TaqMan fast Advanced Master Mix (Thermo Fisher Scientific, no. 4444556) for TaqMan probes, using the CFX‐connect RT‐PCR system. The results were normalised against the expression of the endogenous genes G6PDH, HPRT1 or POLR2B. Only the results normalised against G6PDH are presented, but no differences were observed with HPRT1 or POLR2B.

### 2.7 Statistical Analyses

GraphPad Prism software (version 10.6.1) was used for statistical analyses and graph drawing. Results obtained in the different experiments were analysed with paired Student *t* test, unpaired Mann–Whitney test or with paired one‐way ANOVA test, as appropriate according to the different data set. *p < 0.05, **p < 0.01.*

## 3 Results

### 3.1 PDC*Line Cells‐Based Vaccine Induces Human Naive T Cell Priming and Migrates to Lymph Nodes

PDC*line cells have been used to develop a vaccine platform for the treatment of cancer patients [24, 25]. We first checked the priming of naive T cells by PDC*line cells in vivo in humanised mice. Immunodeficient NOG mice were first injected with CD34+ stem cells, to obtain the development of naive human T cells [29]. Following weekly vaccination with MelA peptide‐loaded and irradiated PDC*line cells, MelA‐specific CD8+ T cells were measured in the blood, and in the spleen of the mice. As shown in Figure 1A, MelA‐specific CD8+ T cells were detected in the blood of two out five vaccinated mice from Day 21. At Day 28, in these two mice, specific T cells represented 5.38% and 1.43% of circulating CD8+ T cells and 0.91% and 0.50% of splenic CD8+ T cells (Figure 1A,B). Since priming of naive T cells involves several cell types and complex signalling pathways in LNs [30], we wondered whether the PDC*line‐based vaccine migrated toward LNs after intravenous injection. Luciferase‐expressing PDC*line were injected into nude mice that had been humanised with human PBMC. Four hours after injection, mice were sacrificed and bioluminescence signal was measured in LNs (Figure 1C,D). Bioluminescence signal was significantly detected in LNs, with a higher signal detected in mesenteric LNs compared to brachial, inguinal or axillary LNs (Figure 1D), demonstrating that the PDC*line cells were able to migrate to LNs.

**FIGURE 1:**
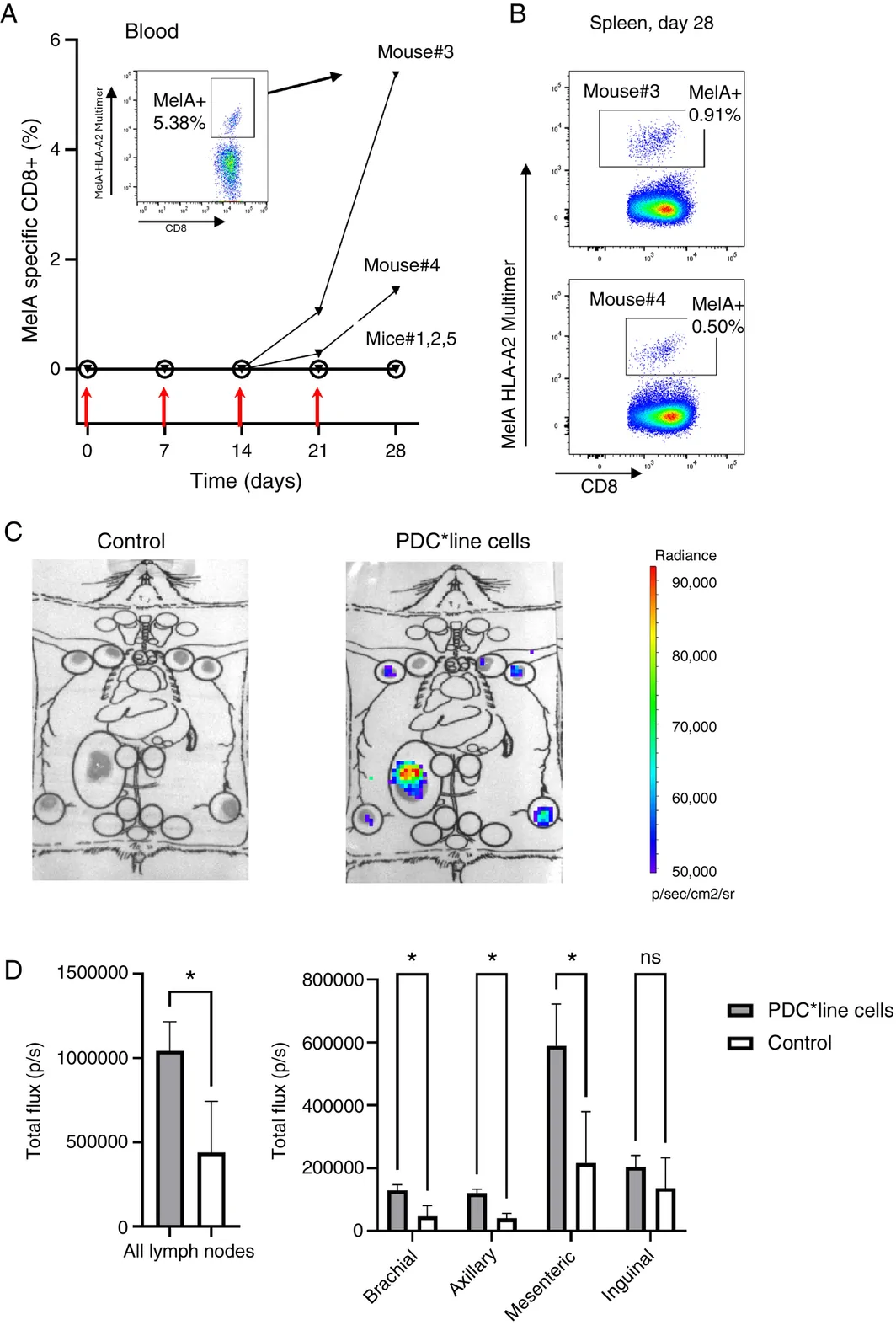
Irradiated PDC*line cells prime human naive antigen‐specific T cells in vivo and migrate to lymph nodes. (A) Kinetics of appearance and example of staining of antigen‐specific CD8+ T cell directed toward MelA peptide among circulating CD8+ T‐ cells in humanised NOG mice following weekly vaccinations with the MelA peptide‐loaded and irradiated PDC*line cells. (B) MelA‐specific CD8+ T cells among gated live human CD45 + CD3 + CD8+ splenocytes of the two responding mice. (C) Example of bioimaging signal of isolated lymph nodes extracted from humanised nude mice 4 h after intravenous injection of luciferase‐expressing PDC*line cells in one injected mouse (right panel) compared to one control mouse (left panel). (D) Total bioluminescence signal measured in all lymph nodes extracted from the 6 humanised nude mice 4 h after injection of luciferase‐expressing PDC*line cells and the three control mice (left panel). Mean and standard deviation of bioluminescence signal measured in isolated lymph nodes derived from the six injected and three control mice (right panel). *p < 0.05 (Unpaired Student's *t* test).

### 3.2 Irradiation of PDC*Line Cells Induces CD62L Shedding

The expression of CD62L was analysed at the surface of resting and irradiated PDC*line cells. CD62L was highly expressed by resting PDC*line cells (> 80% positive cells), and drastically downregulated 4 h after irradiation, as shown in Figure 2A. Of note, cell viability remained high 6 h after irradiation (Figure S2). Then CD62L concentration was measured in whole cell lysates and in cell supernatants. Loss of CD62L in cell lysates was accompanied by an increase of soluble CD62L in cell supernatants, suggesting that irradiation induced CD62L shedding rather than internalisation (Figure 2B,C). This shedding was active at 37°C, but not at room temperature (Figure 2D), suggesting the involvement of enzymatic activity, and was quite at its maximum 2 h postirradiation (Figure 2E). CD62L shedding was dependent on the dose of irradiation, already observed after a 2‐Gy irradiation and almost complete after a 30–60 Gy‐irradiation (Figure 2F). Loss of CD62L was detected as soon as 10 min after irradiation, and reached its maximum after 2 h (Figure 2G).

**FIGURE 2:**
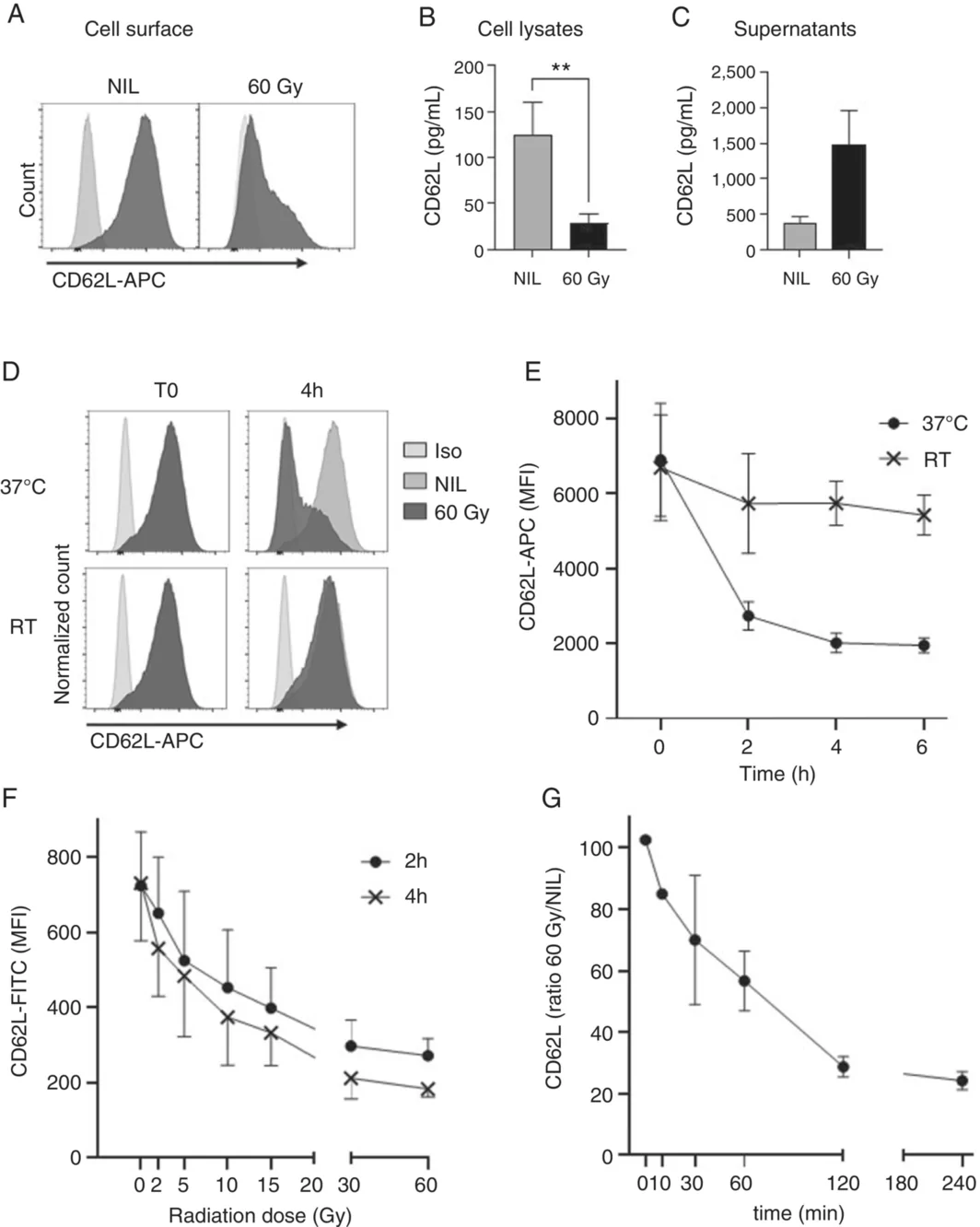
Characterisation of the radiation‐induced CD62L shedding in PDC*line cells. (A) Example of cell surface expression of CD62L (dark histograms) detected by flow cytometry, 4 h after irradiation of PDC*line cells (right panel), compared to nonirradiated cells (left panel). Isotype control is shown in light grey. (B) Measurement of CD62L protein in cell lysates (*n* = 4) and concomitant soluble CD62L in the supernatant (C) obtained from PDC*line cells 4 h postirradiation or not (NIL) (*n* = 2). Soluble CD62L was measured by Human Soluble Protein array. **p < 0.01 (paired Student's *t* test). (D) Representative flow cytometry histogram of CD62L expression in nonirradiated cells (NIL) or following irradiation and incubation for 4 h at room temperature or at 37°C. (E) Kinetics of CD62L expression (mean fluorescence intensity measured by flow cytometry) at the surface of irradiated PDC*line cells over time at room temperature (RT) or at 37°C. Mean with SD from 3 independent experiments. (F) CD62L expression on irradiated PDC*line cells according to the dose of irradiation, after a 2‐ or 4‐h incubation at 37°C. Mean with SD from four independent experiments. (G) Kinetics of CD62L expression over time at the surface of irradiated PDC*line, expressed as the following ratio: MFI of CD62L on irradiated cells/MFI of CD62L on nonirradiated cells * 100. Mean and SD of 1 (10 min), 2 (0 min, 30 min), 3 (60 min) or 4 (120 and 240 min) independent experiments.

### 3.3 CD62L Shedding After Irradiation Is Observed in Primary PDCs, But Not in Other Leukocytes

We then wondered whether primary PDCs and other CD62L‐expressing leukocytes also shed CD62L in response to irradiation. Whole blood from healthy donors was irradiated, and CD62L expression was monitored at the surface of PDCs and of lineage (Lin)‐positive/HLA‐DR‐negative cells (Figure 3A), that mainly comprised T lymphocytes and Natural Killer cells (T + NK cells). CD62L was expressed by 97.5% ± 2.5% of primary PDCs, and by 67.6% ± 13.2% of T + NK cells (Figure 3B,D). As shown in Figure 3B,D, the expression of CD62L at the surface of T + NK cells was reduced in the positive control condition with PMA (50.3% ± 22.5% at 18 h), and was not modified by the irradiation. Conversely, the expression of CD62L was significantly reduced at the surface of primary PDCs when irradiated. While nonirradiated cells retained high expression of CD62L, 4 h after irradiation, CD62L dropped in the positive control condition with PMA (3.3% ± 3.6%), and was expressed by only 73.1% ± 13.5% of PDCs (*p* = 0.004). Eighteen hours after irradiation, CD62L expression dropped to 56.3% ± 30.3% (*p* = 0.05) (Figure 3D). Altogether, these results show that CD62L was also shed following irradiation of primary PDCs, and that this shedding was not a common cellular response to irradiation as T + NK cells kept their CD62L expression in the same conditions.

**FIGURE 3:**
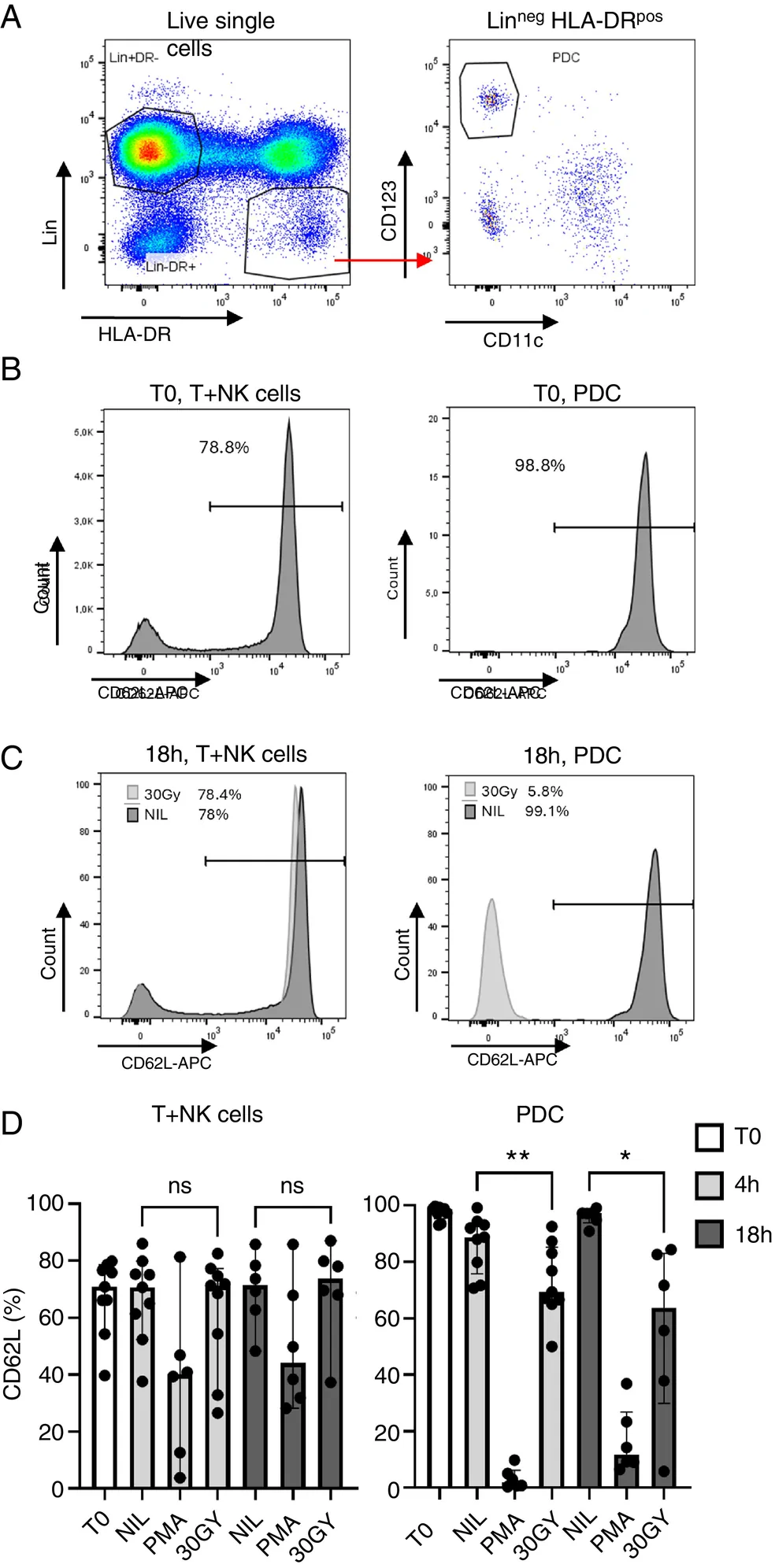
CD62L shedding after irradiation of primary PDCs. (A) Gating strategy for the identification of primary PDC (lin^neg^ HLA‐DR^pos^ CD123^pos^ CD11c^neg^) and T + NK lymphocytes (defined as lin^pos^ HLA‐DR^neg^) cells among whole blood live CD45+ single cells. (B) CD62L expression on primary PDCs and T + NK cells following gating strategy shown in (A). (C) Example of CD62L expression on primary PDCs and on T + NK cells, 18 h postirradiation. (D) Percentage of expression of CD62L at the surface of control, irradiated or PMA‐activated T + NK cells (left panel) and primary PDCs (right panel) after a 4‐ or 18‐h incubation. Mean with SD of 6 independent experiments. *p < 0.05, **p < 0.01 (one‐way ANOVA).

### 3.4 ADAM17, Rather Than ADAM8, Participates in the Irradiation‐Mediated Shedding of CD62L in PDC*Line Cells

In neutrophils, ADAM8 and ADAM17 have been described as CD62L sheddases [15]. ADAM8 and ADAM17 were expressed at the surface of PDC*line cells, and their surface expression was not modified by irradiation (Figure 4A). RT‐qPCR showed a slight increase for ADAM8 and ADAM17 transcripts in irradiated compared to nonirradiated cells (Figure 4B). Inhibitors of ADAM8 and/or ADAM17 were selected: batimastat (for ADAM8 and ADAM17), CGS27023A (ADAM17) and TIMP‐3 (ADAM17). TIMP‐3 or batimastat, but not with CGS 27023A, efficiently inhibited CD62L shedding induced by irradiation (Figure 4C). While only 44% ± 11% of irradiated cells expressed CD62L, CD62L remained expressed by 72% ± 15% and 78% ± 14% of the cells in the presence of TIMP‐3 or batimastat, respectively. A combination of the three chemicals almost completely inhibited CD62L shedding (92% ± 4% of positive cells). Based on these results, preferential involvement of ADAM17 in CD62L shedding can be proposed, as TIMP‐3 that targets ADAM17 but not ADAM8 inhibited CD62L shedding.

**FIGURE 4:**
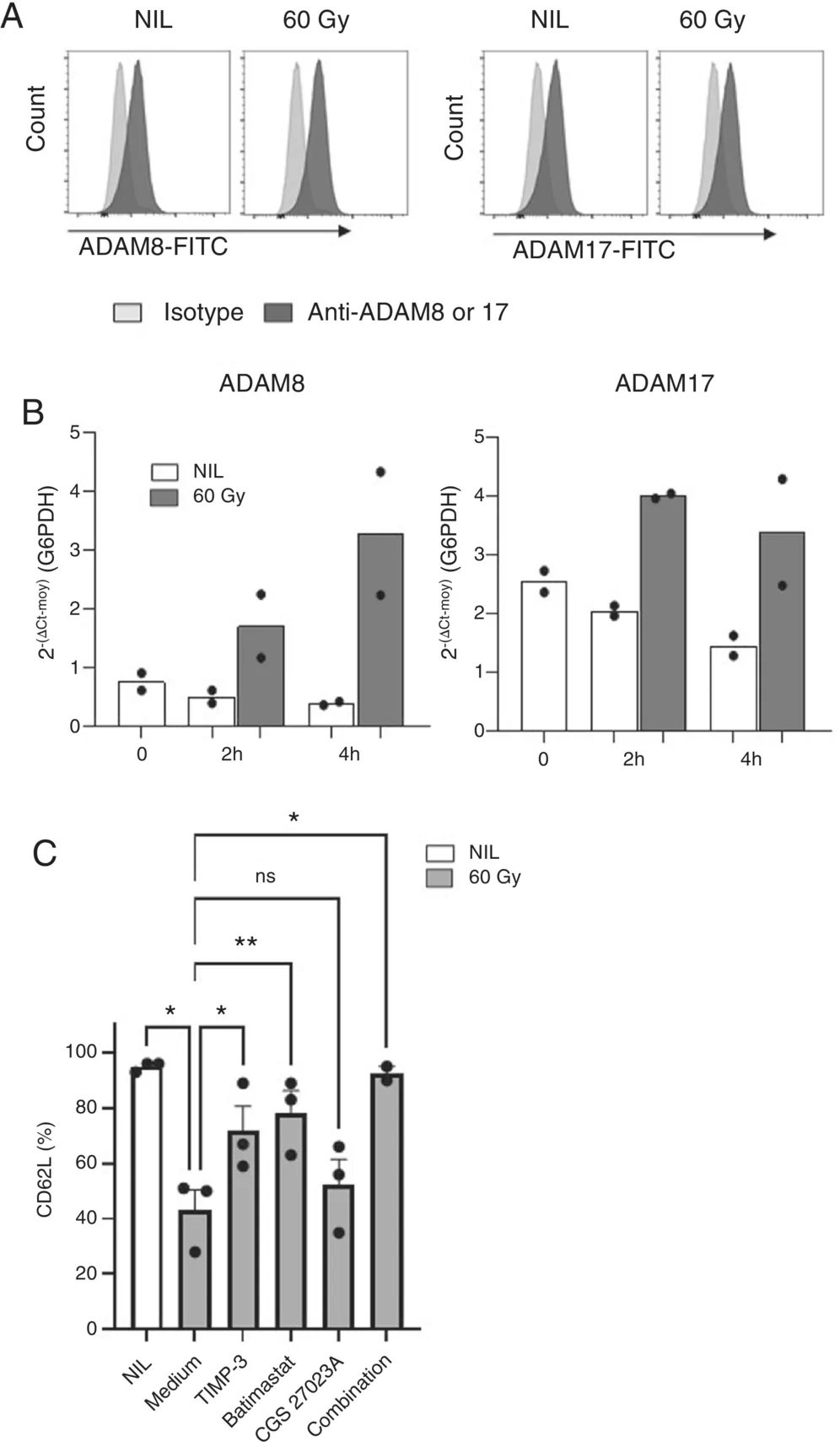
ADAM8 and ADAM17 expression by irradiated PDC*line cells and their involvement in CD62L shedding. (A) Examples of ADAM8 (left panels) and ADAM17 (right panels) expression at the surface of control (NIL) and irradiated PDC*line cells, after a 4‐h incubation. (B) Relative expression (fold change relative to G6PDH) of ADAM8 (left panel) and ADAM17 (right panel) mRNA in control (NIL) and irradiated PDC*line cells after a 2‐ or 4‐h incubation. Mean of two independent experiments. (C) Expression of CD62L at the surface of PDC*line cells measured by flow cytometry 4 h following irradiation. Cells were preincubated or not with chemical inhibitors; batimastat (for ADAM8 and ADAM17), CGS27023A (ADAM17) and TIMP‐3 (ADAM17) alone or in combination. Mean with SD of three independent experiments. *p < 0.05, **p < 0.01 (One‐way ANOVA).

### 3.5 Inhibition of p38 MAPK Overcomes CD62L Shedding on Irradiated PDC*Line Cells

ADAM17 phosphorylation by p38 MAPK, PLK2 or PKCdelta allows the removal of the N‐terminal inhibiting pro‐domain and the activation of ADAM17 protease function (Figure 5A). Oxydative stress, that can be triggered by cell irradiation, is known to activate PLK2 and PKCdelta, while p38 MAPK can be activated following radiation‐induced DNA damage [31, 32]. Inhibitors of these different signalling pathways were used: acetylcystein (inhibitor of oxidative stress), Go 6983 (inhibitor of PKCdelta), adezmapimod (inhibitor of P38MAPK) and ON1231320 (inhibitor of PLK2). Only adezmapimod blocked CD62L shedding (Figure 5B), in a dose‐dependent manner (Figure 5C,D). Altogether, these results suggest that in PDC*line cells, ionising radiation activates the p38 MAPK pathway, and ADAM17, thereby promoting CD62L shedding.

**FIGURE 5:**
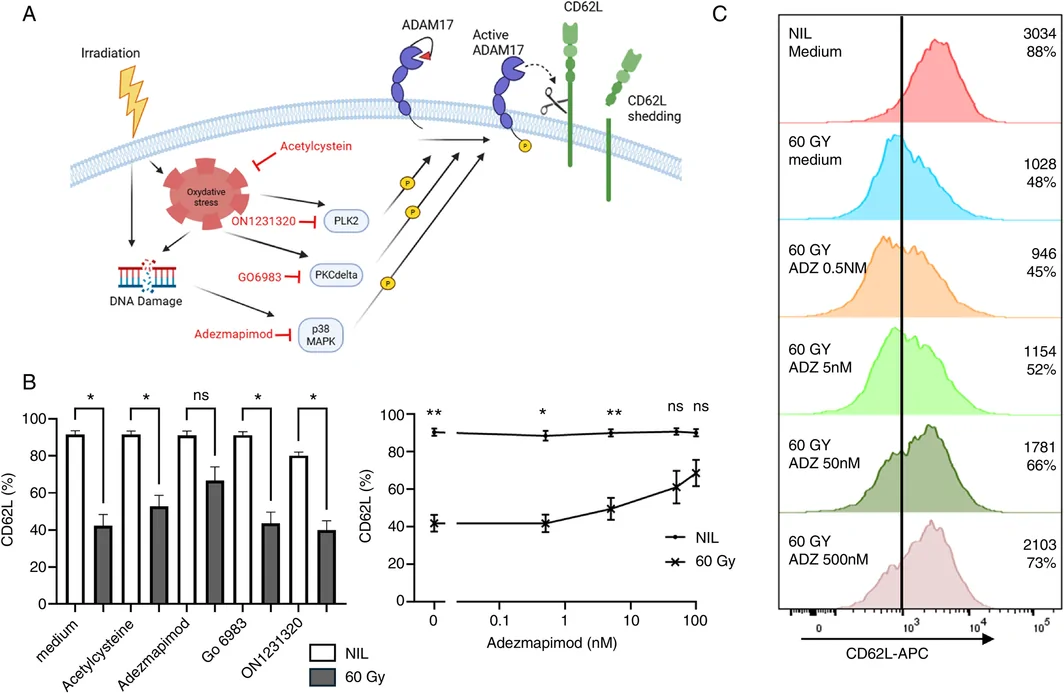
ADAM17 leads to CD62L shedding through p38MAK activation upon irradiation of PDC*line cells. (A) Schematic representation depicting radiation‐induced signalling pathways that could lead to ADAM17 phosphorylation and CD62L shedding (generated with BioRender). (B) Expression of CD62L at the surface of PDC*line cells measured by flow cytometry 4 h following irradiation. Cells were preincubated or not with chemical inhibitors of oxidative stress (Acetylcystein), PLK2 (ON1231320), p38MAPK (Adezmapimod) or PKCdelta (GO6983). Mean with SD of three independent experiments. *p < 0.05 (One‐way ANOVA). (C) Representative experiment of dose‐dependent CD62L shedding after irradiation of PDC*line cells preincubated with increasing concentration of Adezmapimod (ADZ). MFI and percentages of positive cells are shown. (D) Dose‐dependent CD62L shedding after irradiation of PDC*line cells preincubated with Adezmapimod. Mean with SD of four independent experiments. **p < 0.01.

## 4 Discussion

Within the dendritic cell family, PDCs are distinguished by their ability to migrate directly from blood to secondary lymphoid organs, the main sites for T‐cell and B‐cell activation. PDCs traffic from the bloodstream to LNs through HEVs, which involves coordinated chemotaxis, adhesion and cell shape remodelling. In this process, the adhesion molecule CD62L plays a pivotal role in initiating the interaction between lymphocytes and the endothelium of HEVs, mediating their tethering and rolling along vessel walls [33]. Primary circulating human PDCs express numerous chemokine receptors and adhesion molecules, including CD62L [6], which confers them with the capacity to home to lymphoid organs similarly to naive lymphocytes. Despite the well‐established role of CD62L in the TEM of neutrophils, monocytes or lymphocytes [15], its regulation on PDCs remains poorly understood.

Using a therapeutic PDC line model (PDC*line) developed from a patient with BPDCN, we investigated the mechanisms regulating CD62L expression and shedding in PDCs in response to irradiation. BPDCN express CD62L and other trafficking‐related molecules (CD54, CD11a/CD18, CXCR4) similarly to primary PDCs [21]. The derived GMP‐compliant PDC*line, once irradiated and loaded with tumour‐associated peptide is an efficient therapeutic cancer vaccine platform that can triggers specific T‐cell responses [24, 25].

We investigated how the ionising radiation required during the vaccine manufacturing process affected the expression of CD62L. Ionising radiations, such as gamma‐rays or X‐rays, induce severe DNA damage due to DSBs. The sensitivity to DSB‐induced apoptosis varies depending on the cell type, proliferation status and ability to repair DSBs [34]. Following irradiation, PDC*line cells apoptosis is rapidly induced, but cells kept their ability to activate antigen‐specific CD8+ T cells. PDCs sense DNA damage signals through Toll‐like receptor 9 or cGAS–STING, mature [26, 27], and this could participate to the PDC*line vaccine efficacy. In humanised mice, vaccination with the irradiated PDC*line cells activated peptide‐specific CD8+ T lymphocytes, confirming the vaccine capacity to prime naïve T cells in vivo. Bioimaging experiments further demonstrated that viable irradiated PDC*line cells were present in draining LNs 4 h after intravenous injection, strongly suggesting an effective transmigration through HEVs. Of note, the high conservation across species of lymphocyte‐endothelial cell recognition mechanisms involved in lymphocyte homing allowed the study of human leucocytes migration in mice [35].

During TEM, the extracellular portion of CD62L is cleaved, allowing cell migration into the LN paracortex [15], and live imaging studies have shown this cleavage in primary monocytes [16]. We examined the expression of CD62L in PDC*line cells after a 60Gy‐irradiation and observed the rapid and temperature‐dependent disappearance of CD62L from the cell surface, with concurrent detection of its soluble form in the supernatant, suggesting enzymatic shedding. We extended these observations to primary PDCs, in which irradiation similarly induced CD62L shedding, in contrast to T and NK lymphocytes used as controls, underscoring the selective sensitivity of PDCs to radiation‐induced modulation of CD62L expression. The extracellular cleavage site of CD62L (MIKEGDYN) can be targeted by ADAM8 and ADAM17 metalloproteases [19], both expressed in PDC*line cells. The use of selective inhibitors suggested p38MAPK and ADAM17 involvement, as ADAM8 lacks the threonine residue (Thr735) required for p38MAPK‐dependent phosphorylation and activation [36]. Indeed, irradiation‐induced DNA damage leads to activation of the p38 MAPK pathway, its nuclear translocation and subsequent arrest of cell proliferation [31, 32]. Activation of the p38 MAPK pathway also results in phosphorylation and activation of ADAM17, leading to enhanced migratory capacity for irradiated cancer stem cells [37, 38].

Although ADAM10 mRNA was also detected in PDC*line cells (data not shown), several converging lines of evidence argue that ADAM17, rather than ADAM10, is the operative sheddase in our setting. When both enzymes are co‐expressed, ADAM17 is the principal, nonredundant sheddase of CD62L, whereas ADAM10 cleaves this substrate significantly only when ADAM17 is absent or inactivated [39, 40, 41]. Moreover, the shedding we observe is rapid and inducible (detectable at 10 min, maximal at 2 h), a kinetic signature typical of ADAM17, while ADAM10‐dependent CD62L release is comparatively slow and constitutive and is preferentially triggered by calcium influx rather than by the stress/p38 axis engaged here [42]. The neutrophil, the best‐characterised model of CD62L shedding, provides a directly relevant precedent: neutrophils co‐express ADAM17 and ADAM10, yet stimulus‐induced CD62L shedding upon activation is mediated specifically and nonredundantly by ADAM17 both in vitro and in vivo, and is not compensated by other metalloproteases [40, 43], whereas ADAM10 acts constitutively and has been reported not to be a sheddase of L‐selectin upon neutrophil activation [44]. Thus, the mere co‐expression of several candidate ADAMs does not imply redundant function, and our rapid, inducible, p38‐dependent shedding is fully consistent with ADAM17 being the responsible enzyme. While a genetic ADAM17 loss‐of‐function approach would provide formal confirmation, the convergent pharmacological, kinetic and comparative evidence presented here consistently points to ADAM17 as the operative sheddase, and genetic validation represents a natural direction for future work.

As mentioned earlier, CD62L is essential for the initial tethering/rolling of leukocytes on HEVs, facilitating their entry into LNs from the bloodstream. Yet, our data reveal rapid CD62L shedding on irradiated PDCs, prompting the question of how these intravenously injected cells nonetheless home efficiently to LNs. It takes about 1 min for blood to get around the entire human body, and the velocity of blood in LNs HEV capillaries can reach 1571 μm/s [45, 46], allowing T and B lymphocytes to cross HEVs and reach the paracortex within minutes [33, 47]. Thus, irradiated PDCs likely can undergo CD62L‐dependent rolling and enter LNs before complete CD62L release, reconciling this apparent temporal paradox with efficient homing. Our in vivo data establish the temporal feasibility of migration despite shedding: irradiated PDC*line cells reach the LNs within the very time window during which CD62L shedding is actively occurring. However, these results do not allow a causal assessment of the effect of gradual CD62L levels on migration, which would require CD62L overexpression or gene inactivation combined with in vivo migration assays. These findings establish the feasibility and temporal consistency of PDC homing despite CD62L shedding, providing a solid foundation for future causal studies.

The relevance of irradiation‐induced CD62L shedding also depends on the route of clinical administration. Following intravenous administration, PDC*line cells must engage the multistep adhesion cascade on HEVs, in which CD62L‐dependent tethering/rolling is a rate‐limiting first step for blood‐to‐lymph‐node homing; irradiation‐induced CD62L shedding is therefore most pertinent in this setting. In contrast, after subcutaneous administration, cells injected into peripheral tissue reach the draining LNs primarily via afferent lymphatic vessels through CCR7/CCL21‐driven mechanisms, in which CD62L plays a comparatively minor role 33. Although little is known about this route specifically for PDCs, such a mechanism is highly plausible given that PDCs express CCR7 [13]. In this setting, the impact of CD62L shedding on trafficking is therefore expected to be limited.

Altogether, these results reveal that the DNA damage‐induced activation of ADAM17 by the p38 MAPK signalling pathway drives CD62L shedding in PDCs, linking the cellular stress response to the migratory behaviour essential for their immune functions in secondary lymphoid organs.

## Author Contributions

S.P., J.P., L.C. designed research. E.L., J.K., G.V., D.H., J.‐P.M. performed research. D.G., V.J. contributed analytic tools. L.C., J.P., K.L., S.P. analysed data. L.C., J.P., wrote the paper. L.C., J.P., P.S. contributed to discussions that led to the writing of the paper. L.C., J.P., E.L., K.L. prepared figures. All authors participated in the critical revision of the manuscript.

## Funding

This work was supported by the Etablissement Français du Sang, the PDC*Line Pharma, the Wallon Region (Grant no. 7833), and the France Life Imaging (ANR‐11‐INBS‐0006).

## Conflicts of Interest

Joel Plumas is cofunder, shareholder and employee of PDC*line Pharma. Estelle Leplus, Séverine Planel, Guillaume Vayssiere, Jamila Kacher and Karine Laulanier are employees of PDC*line Pharma. Dan Georgess is an employee of TransCure bioServices, Véronique Josserand is an employee of the Optimal Grenoble platform of the Institute for Advanced Biosciences. The other authors declare no conflicts of interest.

## Supporting information


**Figure S1:** Humanisation and gating strategy for MelA‐specific CD8+ T cells identification. (A) Fourteen weeks after CD34+ cell injection, engraftment level was monitored with the analysis of the percentage of human CD45+ cells among total blood leukocytes by flow cytometry (Attune, Life technologies). Histograms show median percentages for vaccinated and control mice at Day 28 postvaccination. (B) Gating strategy for identification of MelA‐specific CD8+ T cells among live human CD45+ CD3+ CD8+ T lymphocytes.

**Figure S2:** Viability of PDC*line cells following 60 Gy‐irradiation over time. (A) Representative experiment of time‐dependent apoptosis after 60‐Gy irradiation of the PDC*line cells. Percentages of early apoptotic cells (AnnV^pos^/7AAD^neg^), apoptotic cells (AnnV^pos^/7AAD^pos^) and necrotic cells (AnnV^neg^/7AAD^pos^) cells were determined on the gated ‘all cells’ population. (B) Kinetics of viability loss of 60‐Gy irradiated PDC*Line cells. Viable cells were defined as AnnV^neg^ 7AAD^neg^ cells.

## Data Availability

The data that support the findings of this study are available from the corresponding author upon reasonable request.

## References

[1] A. C. Villani , R. Satija , G. Reynolds , et al., “Single‐Cell RNA‐Seq Reveals New Types of Human Blood Dendritic Cells, Monocytes, and Progenitors,” Science 356 (2017): eaah4573. 10.1126/science.aah4573 PMC5775029 28428369

[2] E. Segura , “Human Dendritic Cell Subsets: An Updated View of Their Ontogeny and Functional Specialization,” European Journal of Immunology 52 (2022): 1759–1767. 10.1002/eji.202149632 PMC9790408 35187651

[3] N. M. Adams , A. Das , T. J. Yun , and B. Reizis , “Ontogeny and Function of Plasmacytoid Dendritic Cells,” Annual Review of Immunology 42 (2024): 347–373. 10.1146/annurev-immunol-090122-041105 38941603

[4] G. Lui , O. Manches , J. Angel , J. P. Molens , L. Chaperot , and J. Plumas , “Plasmacytoid Dendritic Cells Capture and Cross‐Present Viral Antigens From Influenza‐Virus Exposed Cells,” PLoS One 4 (2009): e7111. 10.1371/journal.pone.0007111 PMC2741572 19771163

[5] T. Di Pucchio , B. Chatterjee , A. Smed‐Sorensen , et al., “Direct Proteasome‐Independent Cross‐Presentation of Viral Antigen by Plasmacytoid Dendritic Cells on Major Histocompatibility Complex Class I,” Nature Immunology 9 (2008): 551–557. 10.1038/ni.1602 PMC2695657 18376401

[6] M. Cella , D. Jarrossay , F. Facchetti , et al., “Plasmacytoid Monocytes Migrate to Inflamed Lymph Nodes and Produce Large Amounts of Type I Interferon,” Nature Medicine 5 (1999): 919–923. 10.1038/11360 10426316

[7] B. Vanbervliet , N. Bendriss‐Vermare , C. Massacrier , et al., “The Inducible CXCR3 Ligands Control Plasmacytoid Dendritic Cell Responsiveness to the Constitutive Chemokine Stromal Cell–Derived Factor 1 (SDF‐1)/CXCL12,” Journal of Experimental Medicine 198 (2003): 823–830. 10.1084/jem.20020437 PMC2194187 12953097

[8] T. S. Mathan , C. G. Figdor , and S. I. Buschow , “Human Plasmacytoid Dendritic Cells: From Molecules to Intercellular Communication Network,” Frontiers in Immunology 4 (2013): 372. 10.3389/fimmu.2013.00372 PMC3825182 24282405

[9] C. Lehmann , M. Lafferty , A. Garzino‐Demo , et al., “Plasmacytoid Dendritic Cells Accumulate and Secrete Interferon Alpha in Lymph Nodes of HIV‐1 Patients,” PLoS One 5 (2010): e11110. 10.1371/journal.pone.0011110 PMC2885422 20559432

[10] B. Malleret , B. Manéglier , I. Karlsson , et al., “Primary Infection With Simian Immunodeficiency Virus: Plasmacytoid Dendritic Cell Homing to Lymph Nodes, Type I Interferon, and Immune Suppression,” Blood 112 (2008): 4598–4608. 10.1182/blood-2008-06-162651 18787223

[11] K. N. Brown , V. Wijewardana , X. Liu , and S. M. Barratt‐Boyes , “Rapid Influx and Death of Plasmacytoid Dendritic Cells in Lymph Nodes Mediate Depletion in Acute Simian Immunodeficiency Virus Infection,” PLoS Pathogens 5 (2009): e1000413. 10.1371/journal.ppat.1000413 PMC2671605 19424421

[12] Y. Gao , B. Majchrzak‐Kita , E. N. Fish , and J. L. Gommerman , “Dynamic Accumulation of Plasmacytoid Dendritic Cells in Lymph Nodes Is Regulated by Interferon‐Beta,” Blood 114 (2009): 2623–2631. 10.1182/blood-2008-10-183301 19652204

[13] S. Seth , L. Oberdörfer , R. Hyde , et al., “CCR7 Essentially Contributes to the Homing of Plasmacytoid Dendritic Cells to Lymph Nodes Under Steady‐State as Well as Inflammatory Conditions,” Journal of Immunology 186 (2011): 3364–3372. 10.4049/jimmunol.1002598 21296980

[14] T. G. Diacovo , A. L. Blasius , T. W. Mak , M. Cella , and M. Colonna , “Adhesive Mechanisms Governing Interferon‐Producing Cell Recruitment Into Lymph Nodes,” Journal of Experimental Medicine 202 (2005): 687–696. 10.1084/jem.20051035 PMC2212867 16147979

[15] A. Ivetic , “A Head‐To‐Tail View of L‐Selectin and Its Impact on Neutrophil Behaviour,” Cell and Tissue Research 371 (2018): 437–453. 10.1007/s00441-017-2774-x PMC5820395 29353325

[16] K. Rzeniewicz , A. Newe , A. Rey Gallardo , et al., “L‐Selectin Shedding Is Activated Specifically Within Transmigrating Pseudopods of Monocytes to Regulate Cell Polarity in Vitro,” Proceedings of the National Academy of Sciences of the United States of America 112 (2015): E1461–E1470. 10.1073/pnas.1417100112 PMC4378423 25775539

[17] D. J. Killock and A. Ivetić , “The Cytoplasmic Domains of TNFalpha‐Converting Enzyme (TACE/ADAM17) and L‐Selectin Are Regulated Differently by p38 MAPK and PKC to Promote Ectodomain Shedding,” Biochemical Journal 428 (2010): 293–304. 10.1042/BJ20091611 20331435

[18] J. Schwarz , S. Schmidt , O. Will , et al., “Polo‐Like Kinase 2, a Novel ADAM17 Signaling Component, Regulates Tumor Necrosis Factor α Ectodomain Shedding,” Journal of Biological Chemistry 289 (2014): 3080–3093. 10.1074/jbc.M113.536847 PMC3908438 24338472

[19] A. Ager , “ADAMs and Ectododomain Proteolytic Shedding in Leucocyte Migration: Focus on L‐Selectin and ADAM17,” Current Immunology Reviews 8 (2012): 103–117.

[20] L. Chaperot , N. Bendriss , O. Manches , et al., “Identification of a Leukemic Counterpart of the Plasmacytoid Dendritic Cells,” Blood 97 (2001): 3210–3217. 10.1182/blood.v97.10.3210 11342451

[21] N. Bendriss‐Vermare , L. Chaperot , M. Peoc'h , et al., “In Situ Leukemic Plasmacytoid Dendritic Cells Pattern of Chemokine Receptors Expression and in Vitro Migratory Response,” Leukemia 18 (2004): 1491–1498. 10.1038/sj.leu.2403452 15284853

[22] L. Chaperot , I. Perrot , M. C. Jacob , et al., “Leukemic Plasmacytoid Dendritic Cells Share Phenotypic and Functional Features With Their Normal Counterparts,” European Journal of Immunology 34 (2004): 418–426. 10.1002/eji.200324531 14768046

[23] L. Chaperot , A. Blum , O. Manches , et al., “Virus or TLR Agonists Induce TRAIL‐Mediated Cytotoxic Activity of Plasmacytoid Dendritic Cells,” Journal of Immunology 176 (2006): 248–255. 10.4049/jimmunol.176.1.248 16365416

[24] J. Charles , L. Chaperot , D. Hannani , et al., “An Innovative Plasmacytoid Dendritic Cell Line‐Based Cancer Vaccine Primes and Expands Antitumor T‐Cells in Melanoma Patients in a First‐In‐Human Trial,” Oncoimmunology 9 (2020): 1738812. 10.1080/2162402X.2020.1738812 PMC7153838 32313721

[25] J. Vansteenkiste , I. Demedts , K. Cuppens , et al., “Innovative Therapeutic Cancer Vaccine PDC*lung01 With or Without Anti‐PD‐1: An Open‐Label, Dose‐Escalation Phase I/II Study in Non‐Small‐Cell Lung Cancer,” ESMO Open 10 (2025): 105844. 10.1016/j.esmoop.2025.105844 PMC12550581 41101155

[26] C. Bode , M. Fox , P. Tewary , et al., “Human Plasmacytoid Dentritic Cells Elicit a Type I Interferon Response by Sensing DNA via the cGAS‐STING Signaling Pathway,” European Journal of Immunology 46 (2016): 1615–1621. 10.1002/eji.201546113 PMC6389263 27125983

[27] A. Krug , S. Rothenfusser , V. Hornung , et al., “Identification of CpG Oligonucleotide Sequences With High Induction of IFN‐Alpha/Beta in Plasmacytoid Dendritic Cells,” European Journal of Immunology 31 (2001): 2154–2163. 10.1002/1521-4141(200107)31:7<2154::aid-immu2154>3.0.co;2-u 11449369

[28] D. Hannani , E. Leplus , D. Laurin , et al., “A New Plasmacytoid Dendritic Cell‐Based Vaccine in Combination With Anti‐PD‐1 Expands the Tumor‐Specific CD8+ T Cells of Lung Cancer Patients,” International Journal of Molecular Sciences 24 (2023): 1897. 10.3390/ijms24031897 PMC9915756 36768214

[29] G. Marodon , D. Desjardins , L. Mercey , et al., “High Diversity of the Immune Repertoire in Humanized NOD.SCID.Gamma c−/− Mice,” European Journal of Immunology 39 (2009): 2136–2145. 10.1002/eji.200939480 19572320

[30] K. Jobin , D. Seetharama , L. Ruttger , et al., “A Distinct Priming Phase Regulates CD8 T Cell Immunity by Orchestrating Paracrine IL‐2 Signals,” Science 388 (2025): eadq1405. 10.1126/science.adq1405 40208984

[31] X. Wang , C. H. McGowan , M. Zhao , et al., “Involvement of the MKK6‐p38gamma Cascade in Gamma‐Radiation‐Induced Cell Cycle Arrest,” Molecular and Cellular Biology 20 (2000): 4543–4552. 10.1128/mcb.20.13.4543-4552.2000 PMC85840 10848581

[32] C. D. Wood , T. M. Thornton , G. Sabio , R. A. Davis , and M. Rincon , “Nuclear Localization of p38 MAPK in Response to DNA Damage,” International Journal of Biological Sciences 5 (2009): 428–437. 10.7150/ijbs.5.428 PMC2702826 19564926

[33] J. P. Girard , C. Moussion , and R. Förster , “HEVs, Lymphatics and Homeostatic Immune Cell Trafficking in Lymph Nodes,” Nature Reviews. Immunology 12 (2012): 762–773. 10.1038/nri3298 23018291

[34] W. P. Roos and B. Kaina , “DNA Damage‐Induced Cell Death by Apoptosis,” Trends in Molecular Medicine 12 (2006): 440–450. 10.1016/j.molmed.2006.07.007 16899408

[35] N. W. Wu , S. Jalkanen , P. R. Streeter , and E. C. Butcher , “Evolutionary Conservation of Tissue‐Specific Lymphocyte‐Endothelial Cell Recognition Mechanisms Involved in Lymphocyte Homing,” Journal of Cell Biology 107 (1988): 1845–1851. 10.1083/jcb.107.5.1845 PMC2115327 3182939

[36] P. Xu and R. Derynck , “Direct Activation of TACE‐Mediated Ectodomain Shedding by p38 MAP Kinase Regulates EGF Receptor‐Dependent Cell Proliferation,” Molecular Cell 37 (2010): 551–566. 10.1016/j.molcel.2010.01.034 PMC4240279 20188673

[37] S. W. Hong , W. Hur , J. E. Choi , J. H. Kim , D. Hwang , and S. K. Yoon , “Role of ADAM17 in Invasion and Migration of CD133‐Expressing Liver Cancer Stem Cells After Irradiation,” Oncotarget 7 (2016): 23482–23497. 10.18632/oncotarget.8112 PMC5029641 26993601

[38] V. Waller , F. Tschanz , R. Winkler , and M. Pruschy , “The Role of EphA2 in ADAM17‐ and Ionizing Radiation‐Enhanced Lung Cancer Cell Migration,” Frontiers in Oncology 13 (2023): 1117326. 10.3389/fonc.2023.1117326 PMC10043294 36998455

[39] S. M. Le Gall , P. Bobé , K. Reiss , et al., “ADAMs 10 and 17 Represent Differentially Regulated Components of a General Shedding Machinery for Membrane Proteins Such as Transforming Growth Factor Alpha, L‐Selectin, and Tumor Necrosis Factor Alpha,” Molecular Biology of the Cell 20 (2009): 1785–1794. 10.1091/mbc.E08-11-1135 PMC2655247 19158376

[40] Y. Li , J. Brazzell , A. Herrera , and B. Walcheck , “ADAM17 Deficiency by Mature Neutrophils Has Differential Effects on L‐Selectin Shedding,” Blood 108 (2006): 2275–2279. 10.1182/blood-2006-02-005827 PMC1895557 16735599

[41] A. Ivetic , H. L. Hoskins Green , and S. J. Hart , “L‐Selectin: A Major Regulator of Leukocyte Adhesion, Migration and Signaling,” Frontiers in Immunology 10 (2019): 1068. 10.3389/fimmu.2019.01068 PMC6527602 31139190

[42] S. M. Le Gall , T. Maretzky , P. D. Issuree , et al., “ADAM17 Is Regulated by a Rapid and Reversible Mechanism That Controls Access to Its Catalytic Site,” Journal of Cell Science 123 (2010): 3913–3922. 10.1242/jcs.069997 PMC2972273 20980382

[43] H. K. Mishra , J. Ma , and B. Walcheck , “Ectodomain Shedding by ADAM17: Its Role in Neutrophil Recruitment and the Impairment of This Process During Sepsis,” Frontiers in Cellular and Infection Microbiology 7 (2017): 138. 10.3389/fcimb.2017.00138 PMC5403810 28487846

[44] T. P. Condon , S. Flournoy , G. J. Sawyer , B. F. Baker , T. K. Kishimoto , and C. F. Bennett , “ADAM17 But Not ADAM10 Mediates Tumor Necrosis Factor‐Alpha and L‐Selectin Shedding From Leukocyte Membranes,” Antisense & Nucleic Acid Drug Development 11 (2001): 107–116. 10.1089/108729001750171353 11334139

[45] U. H. von Andrian , “Intravital Microscopy of the Peripheral Lymph Node Microcirculation in Mice,” Microcirculation 3 (1996): 287–300. 10.3109/10739689609148303 8930886

[46] R. A. Warnock , S. Askari , E. C. Butcher , and U. H. von Andrian , “Molecular Mechanisms of Lymphocyte Homing to Peripheral Lymph Nodes,” Journal of Experimental Medicine 187 (1998): 205–216. 10.1084/jem.187.2.205 PMC2212097 9432978

[47] C. Park , I.‐Y. Hwang , R. K. Sinha , O. Kamenyeva , M. D. Davis , and J. H. Kehrl , “Lymph Node B Lymphocyte Trafficking Is Constrained by Anatomy and Highly Dependent Upon Chemoattractant Desensitization,” Blood 119 (2012): 978–989. 10.1182/blood-2011-06-364273 PMC3271721 22039261

